# Remote Oncology Care: Review of Current Technology and Future Directions

**DOI:** 10.7759/cureus.10156

**Published:** 2020-08-31

**Authors:** Bradley A McGregor, Gregory A Vidal, Sumit A Shah, James D Mitchell, Andrew E Hendifar

**Affiliations:** 1 Oncology, Dana-Farber Cancer Institute, Boston, USA; 2 Oncology, West Cancer Center and Research Institute and the University of Tennessee Health Science Center, Memphis, USA; 3 Oncology, Stanford University School of Medicine, Palo Alto, USA; 4 Radiation Oncology, Northbay Healthcare, Vacaville, USA; 5 Oncology, Cedars-Sinai Medical Center, Los Angeles, USA

**Keywords:** patient reported outcomes, remote monitoring, oncology, implantable medical device, pro, rpm

## Abstract

Cancer patients frequently develop tumor and treatment-related complications, leading to diminished quality of life, shortened survival, and overutilization of emergency department and hospital services. Outpatient oncology treatment has potential to leave cancer patients unmonitored for long periods while at risk of clinical deterioration which has been exaggerated during the COVID19 pandemic. Visits to cancer clinics and hospitals risk exposing immunocompromised patients to infectious complications. Remote patient reported outcomes monitoring systems have been developed for use in cancer treatment, showing benefits in economic and survival outcomes. While advanced devices such as pulmonary artery pressure monitors and implantable loop recorders have proven benefits in cardiovascular care, similar options do not exist for oncology. Here we review the current literature around remote patient monitoring in cancer care and propose the use of reliable devices for capturing and reporting patient symptoms and physiology.

## Introduction and background

Cancer is a leading cause of morbidity and mortality in the United States, with over 1.8 million new cancer diagnoses and more than 600,000 cancer deaths estimated for 2020 [1]. Furthermore, cancer care significantly impacts the overall healthcare system through high rates of emergency department utilization, hospital admission, and costly treatments whose toxicities have potential to diminish quality of life [2-4].

Cancer patients frequently experience local and systemic symptoms causally related to their malignancy. Local symptoms are due to direct complications of the primary tumor or metastases and can include pain, neurological deficits, respiratory symptoms (cough, shortness of breath, hemoptysis) and obstruction (bowel, biliary, airway). Systemic complications include cachexia, paraneoplastic syndromes, electrolyte abnormalities, metabolic alterations, and hematologic changes. These symptoms are a frequent cause of hospitalization. In a series reported by Numico et al., 74% of hospitalizations of cancer patients were for conditions related to tumor involvement, with a minority for workup or treatment-related complications. The most common symptoms at the time of admission were dyspnea, pain, neurological (not specified by authors), fever, and gastrointestinal (vomiting, jaundice) [5]. Identifying and addressing cancer symptom burden prior to the need for admission is recognized as a critical need and has led to increased utilization of patient-reported outcome (PRO) tools discussed in detail below.

In addition to tumor-related morbidity, cancer patients suffer treatment-related complications. Given the myelosuppressive nature of radiation and chemotherapy, anemia, neutropenia, and thrombocytopenia with associated malaise, infection, and bleeding can occur. One of the most serious complications, neutropenic sepsis, is associated with up to 50% mortality rate, average hospital cost of over $49,000, and total expenditures of $1.1 billion per year in the US [6,7]. Less severe though far more prevalent toxicities such as nausea, vomiting, and diarrhea can have a large negative impact leading to volume depletion, metabolic and electrolyte imbalances and renal failure. Newer immunotherapies are associated with immune-related adverse events with unpredictable timing and vagueness of symptoms, making identification and management especially challenging [2-4].

Modern technology can increase patients’ connectivity to the healthcare system through mobile communications and remote physiologic monitoring [8]. Monitoring systems have been used most extensively for cardiovascular diseases such as congestive heart failure (CHF) and arrhythmia detection. For patients that require long-term monitoring for either of these, implantable devices like CardioMEMS™ and implantable loop recorders (ILR) have been shown to decrease rates of hospitalization, improve arrhythmia detection, and lower costs compared with usual care [9,10]. In oncology, while implantable devices are not available, studies have shown that monitoring patient-reported outcomes reduces visits to the emergency department, decreases follow-up costs and improves overall survival [11-14].

Since the outbreak of a novel SARS-CoV-2 in 2019 (Covid-19), the term “social distancing” has entered the common lexicon. However, social distancing has been a mainstay of oncologic care for decades as immunosuppressed cancer patients take precautions to minimize risk of infections. Patients are instructed to eliminate interactions with sick contacts and avoid large gatherings. Yet at the same time, patients attend frequent visits to outpatient cancer centers for clinical evaluations. For a neutropenic patient, each visit poses a risk for infectious exposure. The latter is further magnified since the onset of the Covid-19 pandemic. As a result, the Centers for Medicare and Medicaid services (CMS) has broadened its coverage of Telehealth services under the 1135 waiver authority and coronavirus preparedness and Response Supplementation Appropriation Act, which has significantly expanded the volume of patients receiving care via telemedicine [15]. Though this has reduced oncology patients’ infectious exposure, it has created the dilemma for laboratory and vital sign monitoring for patients at risk for drug-induced toxicities. Tools that facilitate social distancing while maintaining connectivity to the healthcare system and providing objective data for ongoing management are urgently needed [16,17].

## Review

Monitoring systems for oncology

Representative prospective randomized studies of remote and/or electronic PRO in cancer care are summarized in Table 1. PRO-CTCAE™ (Patient-Reported Outcomes version of the Common Toxicity Criteria for Adverse Events) is a validated tool used to monitor and report toxicities related to cancer treatment in clinical trials. Basch et al. reported results from a prospective randomized study from Memorial Sloan Kettering Cancer Center evaluating the efficacy of an online symptom reporting tool [13,14]. A total of 766 patients with advanced solid tumors undergoing systemic cytotoxic chemotherapy were randomized to either an online symptom reporting platform or usual care. In the experimental arm, patients received weekly email prompts to report on 12 common treatment-related toxicities through a web-based portal called Symptom Tracking and Reporting (STAR). A severe or marked change in symptom reporting prompted an email alert to an oncology nurse; summary reports were made available to the treating physician at the time of clinical visits. Over the course of the study, the STAR platform was associated with an improvement in health-related quality of life scores (34% vs 18%, p < 0.001), with fewer visits to the emergency department (34% vs 41%, p = 0.02) compared to standard of care. Median overall survival was also 20% longer in the PRO arm (31.2 months vs 26.0 months, p = 0.04). The authors proposed that the mechanism of improved survival was related to early interventions including active remote symptom management, supportive medications, chemotherapy dose modifications, and referrals for specialty consultation that prevented downstream consequences. Additionally, the PRO group was able to tolerate continuation of chemotherapy for a longer duration (8.2 months vs 6.3 months, p = 0.002).

**Table 1:** Prospective randomized trials of remote patient monitoring and electronic patient-reported outcomes (ePRO) in oncology

Patients	PRO Intervention	Endpoints	Key Findings
766 adult patients with metastatic breast, gynecologic, genitourinary or lung cancers undergoing chemotherapy [13,14]	Self-reporting via Symptom Tracking and Reporting (STAR) on 12 common chemotherapy-related symptoms: appetite loss, constipation, cough, diarrhea, dyspnea, dysuria, fatigue, hot flashes, nausea, pain, neuropathy, and vomiting	Change in health-related quality of life (HRQL) at six months	HRQL at six months improved in more patients in the PRO arm than the usual care arm (34% vs. 18%) and worsened in fewer patients in the PRO arm (38% vs. 53%; P < 0.001)
		Survival at one year	Median overall survival was prolonged in PRO patients (31.2 vs. 26.0 months; P = 0.03)
		Adherence with STAR self-reporting	73% of patients in the PRO arm completed a self-assessment at any given clinic visit
			Fewer patients required emergency department visits in the PRO group (34% vs. 41%; P = 0.02)
			Patients in the PRO arm received systemic chemotherapy for a longer duration than in the usual care arm (8.2 months vs. 6.3 months; P = 0.002)
133 adult patients with Stage IIATxN1 to Stage IV TxNxM+ non-small cell or small cell lung cancer with non-progressive disease after therapy [11,12]	Self-reporting via e-follow-up application (eFAP) in which 12 symptoms are reported weekly	Overall survival	Median survival was longer in the PRO group (22.5 months vs. 14.9 months, HR 0.59 [95% CI, 0.37-0.96]; P = .03)
		Cost of follow-up	€362 reduction in annual surveillance costs in the PRO arm (€941 vs. €1304)
			Performance status at time of relapse was 0 or 1 in 75.9% of the patients in the PRO arm vs only 32.5% in the control arm
358 adult patients with a cancer diagnosis, life expectancy of more than three months and to receive at least three cycles of chemotherapy [18]	Symptom Care at Home (SCH): telephone-based daily symptom reporting managed by nurse practitioners (NP) compared to enhanced usual care with daily phone symptom reporting without NP interaction	Overall symptom severity including number of days with severe, moderate, mild, and no symptoms	SCH patients had significantly reduced symptom severity across all symptoms measured (P < 0.001)
			SCH patients had significantly fewer days with severe (67% less) and moderate symptom days (39% less) compared with enhanced usual care (P < 0.001 for both).
660 adult patients with any cancer diagnosis undergoing a new course of medical or radiation therapy [19]	Patient completion of the Electronic Self-Report Assessment-Cancer (ESRA-C) covering symptoms and quality of life issues (SQLIs) prior to outpatient visits with oncologist. Printouts of ESRA-C data were provided to the oncologist at the time of the ambulatory visit.	Discussion of SQLIs by clinicians and patients	The odds of SQLI being discussed during an ambulatory visit were only increased when the SQLI reporting was beyond a threshold indicating the patient was having a problem. When SQLI exceeded that threshold, the odds ratio for discussion was 1.287 (95% CI, 1.047 – 1.583).
		Clinic visit duration	There was no difference in mean duration of clinic visit (31.7 minutes vs. 30.3 minutes).
		Clinician evaluation of the intervention	The majority of clinicians reported that the ESRA-C system was useful, with nurses rating it the highest.
100 adult patients undergoing thoracotomy for either primary lung cancer or lung metastases [20]	Telephone keypad and interactive voice response (IVR) symptom reporting completed twice per week over four weeks after hospital discharge following surgery. Email alerts sent to advanced practice nurses versus control where nurses received no notification.	Group differences in symptom threshold events	The number of symptom threshold events was approximately 12% less in the intervention group versus the control group (P = 0.003).
		Differences in mean symptom severity between discharge and follow-up	Symptom severity decreased over time for both groups and was not significantly different between the two groups.
95 adult patients with advanced non-small cell lung cancer (NSCLC) [21]	Collection of data from the electronic Lung Cancer Symptom Scale (ECLSS-QL)	Palliative care referrals	Non-significant increase in referrals to palliative care with ECLSS-QL
		Health-related quality of life	No difference with ECLSS-QL
264 adult patients with incurable, symptomatic, solid tumors receiving chemotherapy [22]	Mobile-based Edmonton Symptom Assessment Scale (E-MOSAIC)	Global quality of life (G-QoL) measuring using EORTC-QIQ-c30 at baseline and six weeks	No significant difference in G-QoL between intervention and control arms. Improvement in symptoms in intervention arm (P = 0.003).
752 adult ambulatory patients with any cancer diagnosis starting a new treatment regimen [23]	Electronic symptom and quality of life (SxQOL) screening tool with or without targeted education, communication coaching, and ability to track data over time	Symptom distress as measured using the 15 item symptom distress scale (SDS-15)	Significantly lower SDS-15 scores in the intervention group (P = 0.02). Intervention was strongest in patients older than 50 (P = 0.002).
325 adult patients undergoing therapy for breast or prostate cancer [24]	WebChoice: an internet-based application for patients to monitor symptoms, receive information on self-care, communicate with nurses, and connect with other patients for support	Symptom distress as measured using Memorial Symptom Assessment Scale-Short Form (MSAS-SF)	Significantly improved symptom distress as measured by MSAS-SF in the intervention group (slope estimate, -0.052 [95% confidence interval, -0.101 to -0.004]; t = 4.42; P = .037).
139 adult breast cancer patients undergoing chemotherapy [25]	Mobile application with questionnaire regarding ECOG performance status and 30 preselected adverse events with severity rating	Change in daily functional activity and symptoms over three outpatient visits	Patients assigned to use the mobile application in combination with physician review had no significant decrease in performance status over three visits. Performance status outcomes were not significantly different that patients who used the application without physician review or a control group that did not use the app.
261 adult patients with primary or metastatic hepatobiliary tumors treated with chemoembolization, radioembolization, or surgical resection and 179 family caregivers [26]	Cancer Support System (CaSSY): web-based intervention with written and audiovisual self-management strategies, a bulletin board, and other resources, visits with a care coordinator during a physician’s appointment every two months, and telephone follow-up every two weeks.	Measures of symptom burden	Decreased symptom burden with use of CaSSY system as measured by Functional Assessment of Cancer Therapy – General (FACT-G) (P < 0.05)
		Interleukin (IL)-1a, IL-1b, IL-6, and IL-8 levels, Natural Killer (NK) cell numbers	Reductions were noted in IL-6, IL-1β, IL-1α, and IL-8, and increase in NK cell numbers were observed but were not statistically significant
		Caregiver stress and depression	Caregiver stress was significantly reduced as measured by the Caregiver Quality of Life Index Cancer Scale (P = 0.05)

A similar PRO-based system was tested in the setting of follow-up care for lung cancer. Denis et al. randomized patients with stage IIA or higher lung cancer within three months of previous treatment to either usual care or symptom monitoring using the Sentinel PRO system which involved weekly questionnaires covering 13 symptoms and subsequent automated alerts to the care team for predetermined thresholds in symptom severity or symptom worsening [11]. A total of 133 patients were enrolled from five treatment centers in France; patients with metastatic disease on non-cytotoxic therapy such as tyrosine kinase inhibitors, immunotherapy or antiangiogenic therapy were eligible. The primary endpoint was overall survival. Sixty-three percent had stage IV disease and 17% had a diagnosis of small cell lung cancer. Ten out of 34 surviving patients in the control group were eligible for crossover into the PRO arm. After two years of follow-up, median survival was longer in the PRO group (22.5 months vs. 14.9 months, HR 0.59 [95% CI, 0.37-0.96]; P = .03) with similar results reported after censoring for crossover. Performance status at time of relapse was 0 or 1 in 75.9% of the patients in the PRO arm vs only 32.5% in the control arm. First relapses were detected outside of scheduled visits to the oncologist 72.4% of the time in the PRO group versus 32.5% in the control group (p < 0.001). A pre-specified secondary analysis of cost-effectiveness reported by Lizée et al., demonstrated a €362 reduction in annual surveillance costs in the PRO arm (€941 vs. €1304) [12].

One of the earliest uses of patient-driven technology to monitor outcomes was the Cancer Care Monitor used by the West Clinic, Memphis, TN, in 2003 [27]. Prior to each scheduled oncology visit, patients would report their symptoms on computers and later digital tablets (Patient Care Monitoring) [28]. This validated survey was used to support a web and mobile-based application prospectively applied to postmenopausal women starting new anti-hormonal therapy to assess whether this tool would improve symptom burden and medication adherence [29]. Of significance, this application was able to increase medication adherence at eight weeks, when complimented with weekly reminders (100% vs 75%, p < 0.05).

Additional ongoing prospective randomized trials evaluating remote patient monitoring and patient reported outcomes in oncology are summarized in Table 2. In the United States, the Symptom Management Implementation of Patient Reported Outcomes in Oncology (SIMPRO) study aims to enroll 18,000 patients with thoracic, gastrointestinal, and gynecologic malignancies. Patients randomized to the intervention arm will enter symptom data into the mobile electronic symptom management system (eSyM). The primary outcome is 30-day emergency department treat and release rate as measured through medical record abstraction [30]. An additional ongoing US study, the THRIVE study, is a prospective, randomized trial evaluating a web-enabled application to improve adherence to hormonal therapy in women with breast cancer. The study will randomize 300 patients into three separate arms: 1. a group that receives weekly reminders through the application, 2. a group that receives weekly reminders and tailored feedback, and a usual care group. The primary endpoint is medication adherence as determined using an electronic pillbox [31].

**Table 2:** Ongoing prospective randomized trials of remote patient monitoring and ePRO in oncology ePRO: electronic patient-reported outcome

Study	Patient population	Intervention	Primary outcome measures
The electronic Symptom Management using the Advanced Symptom Management System (ASyMS) Remote Technology (eSMART) [32]	1108 adult patients undergoing chemotherapy for breast, colorectal, or hematologic cancers in five European countries	Electronic symptom reporting using the electronic Symptom Management using the Advanced Symptom Management System (ASyMS)	Measurement of symptom burden, with secondary outcomes including quality of life, supportive care needs, anxiety, self-care self-efficacy, work limitations, cost effectiveness, and changes in clinical practice in response to PRO data
Patient Remote Intervention and Symptom Management System (PRISMS) [33]	222 adult patients undergoing chemotherapy for chronic lymphocytic leukemia (CLL), Hodgkin’s lymphoma, and non-Hodgkin’s lymphoma (NHL) in two Australian hospitals	Symptom reporting via a computer tablet-based software system that prompts patients to enter twice daily data regarding physical and emotional symptoms	Symptom burden due to nausea, mucositis, constipation, and fatigue
The HOPE Trial: Helping Our Patients Excel [34]	110 adult patients with recurrent, incurable gynecologic malignancies	Combined PRO mobile application and wearable activity tracker	Feasibility and acceptability of the HOPE app and the wearable accelerometers Comparison of two wearable accelerometers (Fitbit Zip and Fitbit Charge HR) for use in pilot RCT. Change from baseline in health-related quality of life (comparing patient-reported baseline and post-baseline EuroQoL EQ-5D).
Home Telemonitoring for Patients With Lung Cancer (HTPLC) [35]	70 adult patients admitted to the hospital for lung cancer as primary or secondary diagnosis	Honeywell HomMed Genesis™ DM Remote Patient Care Monitor	Changes in temperature, pulse rate, blood pressure, SpO2 and weight are measured by telemonitor daily over 14 days after hospital discharge
Self-monitoring and Reminder Texts to Increase Physical Activity After Cancer II (SmartPaceII) [36]	44 adult patients with colon or rectal cancer expected to receive at least 12 weeks of chemotherapy	Activity tracking using FitBit™	Fitbit™ wear time Acceptability of the intervention Text message response rate
Symptom Management Implementation of Patient Reported Outcomes in Oncology (SIMPRO) [30]	18,000 adult patients with thoracic, gastrointestinal, or gynecologic malignancies following surgery or scheduled to start a new treatment plan.	Mobile electronic symptom management system (eSyM)	Emergency Department - Treat and Release (EDTR) Rate at 30 days
THRIVE Study [29]	300 adult women undergoing hormonal therapy for breast cancer	Web-enabled application to improve adherence to hormonal therapy	Adherence to hormonal therapy as determined using an electronic pillbox.
eRAPID [37]	504 adult patients receiving chemotherapy for breast, colorectal, or gynecologic cancer	Electronic patient self-reporting of adverse events: patient information and advice (eRAPID).	Quality of life as measured using FACT-G
Karolinska mHealth Study [38]	150 prostate cancer patients scheduled for definitive radiation therapy and 150 breast cancer patients scheduled for neoadjuvant chemotherapy	Daily symptom reporting using a mobile platform on either mobile phone or tablet computer.	Symptom burden, quality of life, health literacy, disease progress, and health care costs

Two large prospective, randomized studies are currently underway in Europe and Australia to further define the indications and benefits of PRO-based systems. The electronic Symptom Management using the Advanced Symptom Management System (ASyMS) Remote Technology (eSMART) study aims to enroll 1108 patients from five European countries undergoing first-line chemotherapy for breast, colorectal, or hematologic cancer [32]. The primary outcome is measurement of symptom burden, with secondary outcomes including quality of life, supportive care needs, anxiety, self-care self-efficacy, work limitations, cost effectiveness, and changes in clinical practice in response to PRO data. A similar study is being undertaken in Australia for hematologic malignancies utilizing the Patient Remote Intervention and Symptom Management System (PRISMS), a computer tablet-based software system that prompts patients to enter twice daily data regarding physical and emotional symptoms [33]. The PRISMS study aims to enroll 222 patients undergoing chemotherapy for chronic lymphocytic leukemia (CLL), Hodgkin’s lymphoma, and non-Hodgkin’s lymphoma (NHL) in two Australian hospitals. The primary outcome focuses on symptom burden due to nausea, mucositis, constipation, and fatigue.

Physiologic monitoring in oncology

Ubiquitous smartphones and miniaturization of sensor and communication technology has led to enormous data gathering enterprises in consumer and healthcare markets. Smartphone accelerometers, GPS tracking, and high-resolution cameras are critical to fitness and wellness applications but also as diagnostic tools such as remote skin cancer detection. These same devices when combined with subjective PRO feedback can be used to guide patient care or provide prognostic data.

Performance status (PS), commonly measured using the Karnofsky or Eastern Cooperative Oncology Group (ECOG) PS scale, is one of the strongest predictors of cancer survival outcomes and risk of treatment toxicity. Patients with a compromised PS have higher risks of morbidity and mortality, but current clinical PS assessments, despite their critical role in clinical trials, are limited by subjectivity and high rates of interobserver variability [39-41]. Many consumer and medical wearables contain three-axis accelerometers and three-axis gyroscopes, providing continuous readings of individual movement which holds significant promise as a means of overcoming the current limitations of physician identified performance status. Gresham et al. evaluated the feasibility of using activity data from FitBit Charge HR® wearable devices as a surrogate for PS in 37 patients with cancer, 92% of whom had stage IV disease [42]. They reported high correlations between average daily step counts and ECOG-PS (r = 0.63); each increase in daily step count by 1000 was associated with a significant decrease in adverse events, hospitalization, and hazard for death. Strong correlation was also reported between these activity metrics and PRO data. Subsequent systematic review of published studies evaluating the use of activity tracking in cancer care identified 41 trials including active cancer patients and those undergoing follow-up and survivorship care [43]. Most trials included breast cancer patients (65%) and focused on exercise (54%) or behavioral (29%) interventions. Twelve trials evaluated daily step counts, and the reported steps per day were slightly lower in patients on active cancer therapy (2885 to 8300 steps/day) compared to survivors (4660 to 11,000 steps per day). No studies included implantable monitoring devices.

Current technology limitations

While PRO platforms and wearable technology have potential to improve care, implementation in the clinic faces many challenges. Web-based PRO platforms rely on patient engagement, which can be increasingly problematic in an aging cancer population with less familiarity with technology. Furthermore, waiting for patients to report symptoms lacks a proactive preventative solution.

With regards to wearable technology, most studies demonstrate feasibility, however patient adherence remains a major limitation. Seventeen of the studies included in the Gresham systematic review reported adherence data, but adherence was defined differently in most studies [43]. The most commonly reported adherence metric was three consecutive days of activity tracking (range: 3-7 consecutive days), with a valid wear time as 5 to 10 hours per day. These definitions of adherence leave large gaps in time where no data is gathered or reported, limiting the ability to produce high fidelity analytics and accurate diagnostic tools. Additionally, for cancer patients facing several months of therapy, three to seven consecutive days of activity tracking does not represent reliable monitoring through the duration of treatment and follow-up.

Dreher et al. demonstrated similar adherence limitations in a study of FitBit® use in breast cancer patients, stating, “Adherence to wearing the Fitbit was low, with 16.9% of patients never syncing their device.” For patients who did sync their devices, the median number of valid activity tracking days (defined as > 10 hours of use) during the 9-month duration of the study was only 44.5% (median = 39.6%, range 0% - 100%) [44]. Even the much-publicized Apple Watch® atrial fibrillation study, a population consisting of Apple Watch® owners, showed poor adherence with only 21% of those who received an irregular pulse notification initiating the first indicated visit [45]. The studies utilizing wearable technology in oncology care demonstrate the importance of objective data gathering, but also highlight the limitations of systems that rely on patient adherence. Alternatively, the studies involving cardiac referenced in the introduction show significant benefits associated with the reliable and continuous monitoring from implantable sensor technology, including disease control, survival, and economic endpoints.

Future directions

Representative ongoing early phase or pilot studies evaluating emerging digital technology in cancer care are summarized in Table 3. Emerging research shows benefits in outcomes and costs of cancer care through use of remote monitoring technology especially electronic patient reported outcomes (ePRO). However, broad clinical adoption has been limited by a lack of commercially available oncology specific solutions, concerns about reimbursement, and limitations associated with low patient adherence. Several companies offer products addressing this need including software and mobile technology startup companies such as Noona (acquired by Varian Medical Systems, Palo Alto, CA), Kaiku Health, and Navigating Cancer, all offering ePRO solutions. Oleena provides a physician prescribed digital therapeutic that integrates ePRO features for oncology. Vital Connect, VivaLnk, and Current Health integrate physiologic data from wearable and in-home connected devices for oncology and other acute or chronic diseases (Table 4).

**Table 3:** Select ongoing early phase studies of emerging technology for remote patient monitoring and ePRO for oncology ePRO: electronic patient-reported outcome

Sponsor	Diagnoses	Estimated Enrollment	Primary Outcome	Technology and Data Gathered
Massachusetts General Hospital	Gastrointestinal Malignancies	75 patients	Feasibility of remote electronic patient monitoring defined as participant use of monitoring device at least 50% of the time within two weeks of enrollment	Wearable vital sign monitor that communicates with patients’ mobile device, data presented on care team dashboard
Gaido® and the Guthrie Clinic	Adult cancer patients receiving chemotherapy	30 patients	Feasibility of Gaido intervention with analyses of utilization, wear time, and completion of PRO over three weeks of enrollment	Gaido® system with Biovotion Everion® wearable device for remote vital sign collection, manual entry of temperature and blood pressure
Sidney Kimmel Cancer Center at Thomas Jefferson University	Head and neck cancer patients undergoing radiotherapy or chemoradiotherapy	41 patients	Percentage of time using wearable technology, compliance defined as wearing device 19/24 hours (80% daily use) for 70% of the days under treatment	FitBit® Charge 3 and online PRO
Augusta University	Adult patients with acute myeloid leukemia (AML) and are candidate for high-dose cytarabine	30 patients	Change in number of ICU admissions and incidence of sepsis compared with historical cohort	Continuous remote temperature monitoring device
Duke University	Adult patients with metastatic cancer	100 patients	Subject participation over 12 months	Noona® ePRO system
AstraZeneca	Unresectable Stage III non-small cell lung cancer	75 patients	Total number of confirmed pneumonitis cases by grade & number of cases identified early through mobile technology in patients receiving darvalumab	Multiparametric mobile technology collecting patient reported outcomes, vital signs, and respiratory function including a spirometer, an armband, and a tablet to collect data.
University of Michigan	Pediatric patients (age 5 or older) eligible for CAR T-cell therapy	30 patients and 30 caregivers	Percentage of caregivers that log on to BMT Roadmap at least once per day for four of seven days while patient is in the hospital and feasibility of implementing the full system including activity tracking in pediatric population	Mobile tablet for utilization of the BMT Roadmap information system and wearable activity tracker
Washington University School of Medicine	Non-metastatic malignancy of the thorax and planning treatment with radiotherapy with or without chemotherapy	50 patients	Percentage of invited symptom reports completed and percentage of questions completed within each invited symptom report [treatment through 90 days of follow-up (estimated to be five months)]	Noona® ePRO system

**Table 4:** Select companies commercializing technology for remote patient monitoring or ePRO in oncology ePRO: electronic patient-reported outcome

Company	Stage	Target Market	Technology
Apple	Commercial	Consumer wellness	Wearable smart watch (Apple Watch®) and mobile phone (iPhone™)
CareVive	Commercial	Oncology practices	Treatment planning and clinical trial matching, ePRO reporting, and survivorship care planning
Current Health	Commercial	Hospitals and oncology practices	Wearable device for vital sign monitoring and data analytics for early detection of complications
FitBit	Commercial	Consumer wellness	Wearable activity tracker and smart watch (Versa™ and Charge™)
Kaiku Health	Commercial	Oncology practices (Europe)	Mobile PRO reporting and data analytics
Navigating Cancer	Commercial	Oncology practices	Digital patient care management, remote PRO monitoring, and patient engagement
Noona	Commercial and clinical trials	Oncology practices	Mobile PRO reporting platform
Oleena	Commercial	Oncology practices	Digital therapeutic/prescribed mobile app with ePRO reporting and symptom management
Oncodisc	Preclinical	Oncology practices and hospitals	Remote oncology care platform with mobile ePRO and vital sign data from intelligent implantable vascular access device
Vital Connect	Commercial	Hospitals	Wearable patch and data reporting platform (VitalPatch™)
VivaLnk	Commercial	Healthcare providers	Wearable vital sign monitoring patch and reporting dashboard

A preferred solution would securely and reliably gather digital physiologic data without requiring patient activation, much like pulmonary artery pressure monitors and implantable loop recorders in cardiology. Additionally, such a system would contain mobile patient engagement and ePRO tools that could be tied to existing reimbursement framework facilitating rapid adoption. Powerful analytic tools including machine learning and artificial intelligence could then be applied to identify early signs of common complications and provide individual patient health and risk profiles, allowing oncologists to make more informed and personalized treatment recommendations. The use of an implantable device in oncology has already been explored, as oncologic cardiologists at MD Anderson Cancer Center have utilized implantable loop recorders and CardioMEMS® to monitor patients at high risk of complications from cardiotoxic systemic therapy, but data regarding safety and efficacy is lacking [46]. To our knowledge, Oncodisc, a San Francisco-based medical technology start-up, is the only company developing implantable monitoring technology and mobile ePRO solutions for oncology. The Oncodisc device, an intelligent implantable vascular access port, takes advantage of existing oncology workflow and a common minimally invasive procedure with established reimbursement.

## Conclusions

The current Covid-19 pandemic has highlighted the need for reliable connected systems to facilitate home care while reducing hospitalizations and clinic visits, especially in immunocompromised cancer patients. Early studies show significant benefits to PRO-based systems, including lower costs and prolongation of survival. Further advances in sensor technology and mobile communications hold great promise for improving cancer outcomes while at the same time reducing costs. However, widespread adoption has been hampered by a lack of commercially available solutions. To that end, implantable physiologic sensor systems and associated data analytic tools, akin to those used in cardiovascular care, should be researched for oncology.
